# Medical Classifications and Terminologies

**DOI:** 10.1007/978-3-319-78503-5_5

**Published:** 2018-05-15

**Authors:** Hercules Dalianis

**Affiliations:** DSV-Stockholm University, Kista, Sweden

## Abstract

This chapter presents the different medical classifications and terminologies as ICD diagnosis codes, SNOMED CT, MeSH, UMLS, ATC etc.

This chapter will present and discuss an important part of clinical text mining, namely the medical classification systems. Medical terminologies, classification systems and available controlled vocabularies are used in healthcare to report, administer, classify and explain diseases and treatment, including medication. In this chapter the history of the medical classification system ICD diagnosis codes will also be told, followed by a description of the more extensive and modern SNOMED CT. For classification of medical literature Medical Subject Headings (MeSH) is used. UMLS developed specifically for mapping between different terminologies and consists of several other terminologies.

ICD-10 is available in several languages, SNOMED CT in some fewer languages and MeSH for even fewer languages. Anatomical Therapeutic Chemical Classification (ATC) codes are used to describe drugs and their chemical components and available in several languages.

We can consider the SNOMED CT and ICD-10 terminologies as the "new" Greek and Latin of medicine. For example, when a ICD-10 diagnosis code such as *J12* is mentioned, everyone knowledgeable in ICD-10 knows that this means the disease *pneumonia* in English, even if the disease is called *lunginflammation* in Swedish.

Other important standards and codes to have knowledge about are *Unstructured Information Management Architecture (UIMA)*, *Fast Healthcare Interoperability Resources (FHIR)*, *Health Level 7 (HL7)* and *OpenEHR*. Many of these classifications have mapping tables between them to perform interoperability. Finally, the matching and mapping of terminologies to clinical text for expanding terminologies will be described.

H. Dalianis, *Clinical Text Mining*, https://doi.org/10.1007/978-3-319-78503-5_5

## 5.1 International Statistical Classification of Diseases and Related Health Problems (ICD)

ICD stands for *International Statistical Classification of Diseases and Related Health Problems*, but is usually shortened to *International Classification of Diseases*. ICD originates from several early classification systems from the eighteenth century. One is from *Genera morborum*, 1763, the "catalogue of diseases", created by Carl Linnaeus, who is also the father of the classification system for naming organisms. The other source is *Nosologia methodica*, the "disease classification", published by François Boissier de Sauvages de Lacroix in 1763. Linnaeus and Sauvages were good friends and influenced each other.

Significant contributions to the classification of diseases were made by William Farr in 1839, after he was appointed Compiler of Abstracts for the English Registration Act, a law passed on registering the cause of death in the population.

When Florence Nightingale, the social reformer and statistician, and the founder of modern nursing, returned to England from the Crimean War in 1860, she emphasised the importance of a proper statistical classification system for diseases. Jointly with William Farr she worked on the technical problems of the classification system.

Jacques Bertillon was a French physician and statistician who introduced the Bertillon Classification of Causes of Death that is considered to be the predecessor to ICD. Bertillon's system was adopted in 1899 by several countries (Moriyama et al. 2011).

ICD-1, the first revision of the International Statistical Classification of Diseases was published in 1900 (in use 1900–1909): ICD-10, the tenth revision was published in 1986, and has been in use since 1995; however, in the USA they still use ICD-9, the ninth revision.

ICD-10 is the latest revision of ICD-9 and is available in the six official languages of the World Health Organization (WHO), which are Arabic, Chinese, English, French, Russian and Spanish, and in 36 other languages including Swedish. ICD contains 32,000 different diagnosis codes divided into 22 chapters or groups.

ICD coding is used both for medical and administrative purposes. For the clinical personnel to classify diseases and know what type of disease a patient has, but also for administrative purposes such as economical planning and statistics for healthcare.

The 22 chapters are the general foundation, going down to more specific subchapters then more and more specific diseases are observed. For example, the three characters J12 mean *viral pneumonia*[1] at a very basic level. Each three-character level can be extended with up to four characters to provide a more specific definition. The first three characters are separated from the following four characters with a period. This example contains a two-character extension 81, giving J12.81

[1]ICD-10, J12 Viral Pneumonia, http://www.icd10data.com/ICD10CM/Codes/J00-J99/J09-J18/J12-. Accessed 2018-01-11.

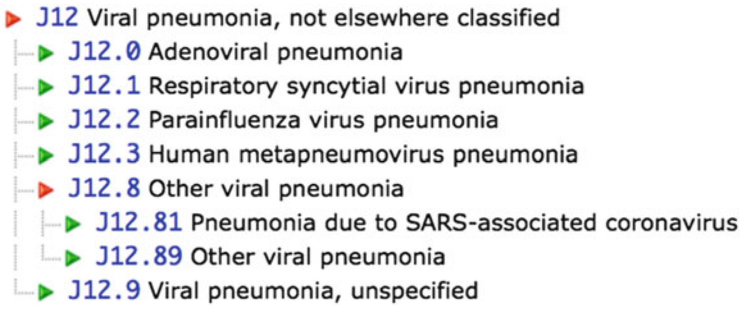

**Fig. 5.1** Hierarchy of the ICD-10 code for J12 Viral pneumonia which means *pneumonia due to SARS-associated corona virus*; see Fig. 5.1 for the hierarchy of the ICD-10 codes. The final two characters' for the position 6 and 7 signify the increased specificity or lateral position of the disease.

### *5.1.1 International Classification of Diseases for Oncology (ICD-O-3)*

There is a special version of ICD, the *International Classification of Diseases for Oncology (ICD-O-3)*,[2] which also is used to code pathology reports for cancer. Pathology reports are also coded using SNOMED CT. ICD-O-3 contains information about the topology and morphology of the cancer. Topology describes the anatomical site of origin, which is where the tumor is situated in the body, and the morphology describes the cell type (histology), stage or behaviour of the tumor (malignant or benign) and number of tumors or metastases.

## 5.2 Systematized Nomenclature of Medicine: Clinical Terms (SNOMED CT)

SNOMED CT stands for the *Systematized Nomenclature of Medicine-Clinical Terms* and originates from two earlier classification systems called *Systematized Nomenclature of Pathology (SNOP)* from 1965 and the United Kingdom's National Health Service Clinical Terms Version 3 (previously known as the Read Codes). SNOP was created to support American pathologists to classify the pathological observations in the categories etiology, morphology, topography and function. SNOMED CT was released in 2002 (Moriyama et al. 2011).

[2]International Classification of Diseases for Oncology (ICD-O-3), http://codes.iarc.fr. Accessed 2018-01-11.

SNOMED CT is available in US English, UK English, Argentine Spanish, Danish and Swedish. Translations into French, Dutch, Lithuanian and several other languages are underway (IHTSDO 2016). SNOMED CT is a clinical, hierarchical terminology containing medical terms and their relations as well as synonyms, including over 320,000 terms. SNOMED CT contains clinical findings (symptoms), disorders (diagnoses), procedures, body structures, organisms etc. Each concept, description, and relationship has a SNOMED identifier that can have up to 18 digits.

Likewise, if we look at SNOMED CT we can identify the general disorder number 233604007, as the disorder *pneumonia* expressed in English, see Fig. 5.2, where also 35 children or subclasses of pneumonia can be seen. However, it is worth mentioning that physicians still might interpret the ICD-10 codes differently, both intra-language wise and inter-language wise.

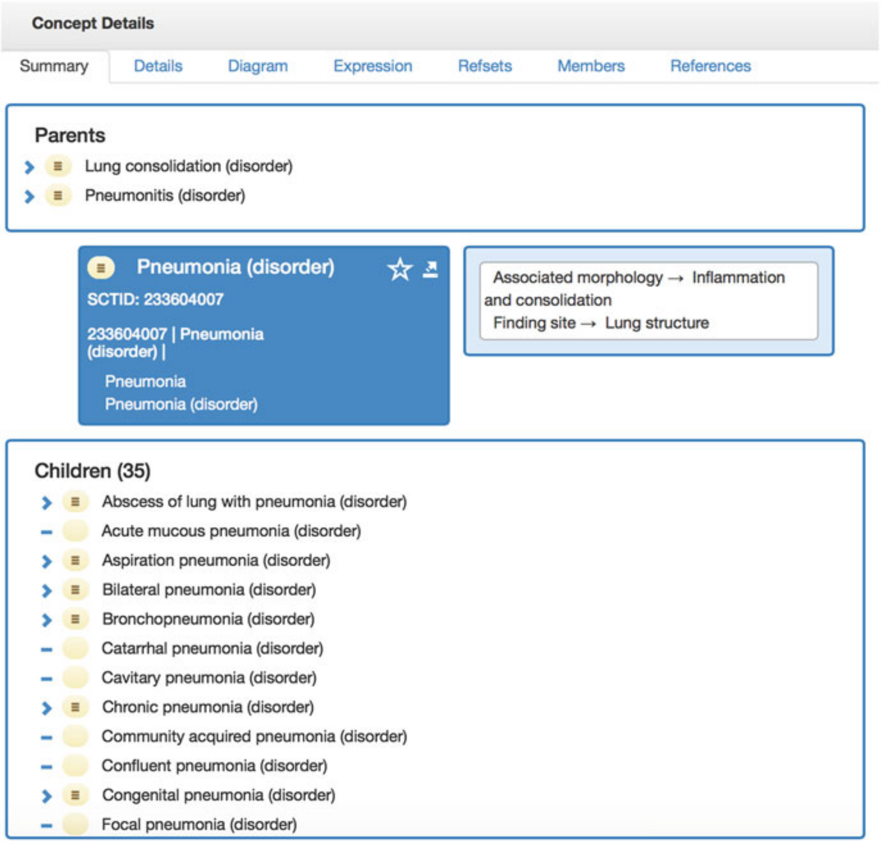

**Fig. 5.2** Hierarchy of the SNOMED CT code for Pneumonia using the IHTSDO SNOMED CT Browser (IHTSDO SNOMED CT Browser, http://browser.ihtsdotools.org/?perspective=full&conceptId1=233604007&edition=en-edition&release=v20160731&server=http://browser.ihtsdotools.org/api/snomed&langRefset=900000000000509007. Accessed 2018-01-11)

ICD-10 has a longer history than SNOMED CT and is widely used and well known, while SNOMED CT is less well known. Both terminologies can be used for cross-language information retrieval but also as plain terminologies for various natural language preprocessing steps. In Chap. 10 these preprocessing steps will be described. SNOMED is a hierarchical system with inheritance and is more expressive than ICD-10, but SNOMED is considered to be more difficult to use.

## 5.3 Medical Subject Headings (MeSH)

MeSH stands for Medical Subject Headings, and is a controlled vocabulary for indexing journal articles and books within life sciences. MeSH was created and is updated by the United States National Library of Medicine (NLM).[3] MeSH is available in over 14 languages.[4]

MeSH is used to categorise publications for libraries, but also to retrieve publications using the MeSH terms. Since MeSH is available in several languages one use cross lingual information retrieval can be used, for example to search in one language and retrieve information in another language.

The 2016 version of MeSH contains in total of 87,000 entry terms (synonyms) to find 27,883 descriptors or subject headings categorised in 16 super headings.[5]

MeSH contains a tree structure from the most general concept to the most specific. If using a MeSH browser different parts of the tree can be browsed, see Fig. 5.3 where the disorder Pneumonia is displayed. The Swedish version of MeSH is a translation of the American-English version, but lacks the amount of synonyms available in English.

## 5.4 Unified Medical Language Systems (UMLS)

UMLS stands for Unified Medical Language Systems[6] and is only available in English. Its purpose is to support mapping between various terminologies. UMLS contains several million concepts stemming from hundreds of bio(medical) vocabularies, such as ICD-10, MeSH and SNOMED CT as well as medical abbreviations. Liu et al. (2002) extracted 163,666 abbreviations full form pairs from UMLS. To read more about UMLS see Humphreys et al. (1998).

[3]US National Library of Medicine, https://www.nlm.nih.gov/mesh/. Accessed 2018-01-11.

[4]MeSH Translations, https://www.nlm.nih.gov/mesh/MTMS_MeSH.html. Accessed 2018-01-11.

[5]MeSH, https://en.wikipedia.org/wiki/Medical_Subject_Headings. Accessed 2018-01-11.

[6]UMLS, https://www.nlm.nih.gov/research/umls/. Accessed 2018-01-11.

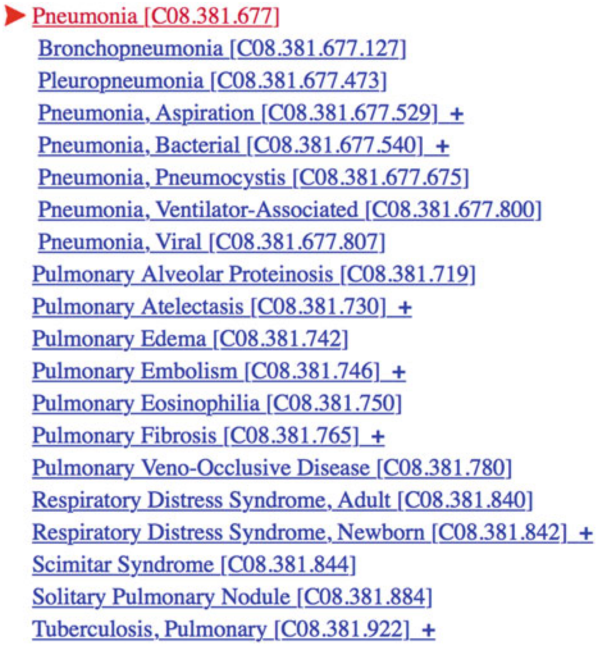

Pneumonia [C08.381.677]
Bronchopneumonia [C08.381.677.127]
Pleuropneumonia [C08.381.677.473]
Pneumonia, Aspiration [C08.381.677.529] +
Pneumonia, Bacterial [C08.381.677.540] +
Pneumonia, Pneumocystis [C08.381.677.675]
Pneumonia, Ventilator-Associated [C08.381.677.800]
Pneumonia, Viral [C08.381.677.807]
Pulmonary Alveolar Proteinosis [C08.381.719]
Pulmonary Atelectasis [C08.381.730] +
Pulmonary Edema [C08.381.742]
Pulmonary Embolism [C08.381.746] +
Pulmonary Eosinophilia [C08.381.750]
Pulmonary Fibrosis [C08.381.765] +
Pulmonary Veno-Occlusive Disease [C08.381.780]
Respiratory Distress Syndrome, Adult [C08.381.840]
Respiratory Distress Syndrome, Newborn [C08.381.842] +
Scimitar Syndrome [C08.381.844]
Solitary Pulmonary Nodule [C08.381.884]
Tuberculosis, Pulmonary [C08.381.922] +

**Fig. 5.3** Part of the MeSH tree of Pneumonia. In this example the numerical coding of the MeSH descriptors (MeSH pneumonia entry, https://www.nlm.nih.gov/cgi/mesh/2016/MB_cgi?mode=&term=Pneumonia&field=entry. Accessed 2018-01-11) can be observed

## 5.5 Anatomical Therapeutic Chemical Classification (ATC)

Each existing drug is represented with an ATC code in the Anatomical Therapeutic Chemical (ATC) Classification[7] which describes each drug with a specific letter and number. The principle of the classification is the active ingredients of the drugs, the organ and structure the act on and their therapeutic, pharmacological and chemical properties. The classification was released in 1976 and is administered by the World Health Organization Collaborating Centre for Drug Statistics Methodology (WHOCC).[8]

ATC codes are structured in five levels. The top level contains the use of the drug, divided in 14 main groups. The second level is the pharmacological and therapeutic subgroup, the third and fourth levels are the chemical, pharmacological and therapeutic subgroups and the fifth level is the chemical substance. The second to fourth levels are used to identify the pharmacological subgroup, see Fig. 5.4 for an example on an ATC code structure.

[7] ATC classification, http://www.whocc.no/atc_ddd_index/.

[8] WHOCC, http://www.whocc.no/atc_ddd_methodology/history/. Accessed 2018-01-11.

| | |
|---|---|
| A | Alimentary tract and metabolism (1st level, anatomical main group) |
| A10 | Drugs used in diabetes (2nd level, therapeutic subgroup) |
| A10B | Blood glucose lowering drugs, excl. insulins (3rd level, pharmacological subgroup) |
| A10BA | Biguanides (4th level, chemical subgroup) |
| A10BA02 | metformin (5th level, chemical substance) |

**Fig. 5.4** The ATC code structure for the chemical substance *metformin* showing its use in lowering glucose for diabetic patients (ATC, http://www.whocc.no/atc/structure_and_principles/. Accessed 2018-01-11)

Drugs are divided in different groups depending on the chemical substance, their therapeutic effect and their pharmacological group.

## 5.6 Different Standards for Interoperability

### *5.6.1 Health Level 7 (HL7)*

*Health Level 7 (HL7)*[9] is the name of a set of standards for the interoperability between different systems in healthcare, for transferring data between patient records systems, and also between patient records systems and laboratory systems or billing systems. See also Health Level Seven International.[10]

#### Fast Healthcare Interoperability Resources (FHIR)

*Fast Healthcare Interoperability Resources (FHIR)*,[11] is a standard within HL7 for sharing data from an electronic patient record system. FHIR communicates an API via a web interface such as http- and https-protocols, JSON, Cascading Style Sheets and also Java.

[9] HL7, https://en.wikipedia.org/wiki/Health_Level_7. Accessed 2018-01-11.

[10] HL7 International, http://www.hl7.org/. Accessed 2018-01-11.

[11] FHIR, https://en.wikipedia.org/wiki/Fast_Healthcare_Interoperability_Resources. Accessed 2018-01-11.

### 5.6.2 OpenEHR

*OpenEHR*[12] is a standard for interoperability specifically between different electronic patient record systems. In Chen and Klein (2007) the open Java interface is described, and in Chen et al. (2009) an experiment performing a bi-directional conversion between the openEHR archetype format to the COSMIC template format is also presented. COSMIC is one of the major electronic patient record systems in Sweden, from Cambio Healthcare Systems. See also the OpenEHR organisation.[13]

UIMA is a special IBM standard for content analytics, see Sect. 8.5.

### 5.6.3 Mapping and Expanding Terminologies

Medical terminologies and classifications are very useful tools for clinical text mining. They can be used for identifying the semantic meaning of lexical concepts in clinical text. For example, is the concept a disorder, a symptom or a body part? The identification of the semantic meaning can support directly the understanding of the clinical text, but the semantic tagging can also be used as features for machine learning.

One other important use of terminologies is to expand the concept with one or more synonyms. This is valuable for identifying and expanding abbreviations and acronyms in clinical text but also for mapping concepts to other terminologies. However, many of the terminologies and classifications are manually made and do not cover medical subdomains. A shared task in this domain is described in Suominen et al. (2013).

Part of the mapping process is to apply computational linguistic methods such as lemmatisation, stemming and compound splitting, see Sect. 7.3.

To create resources manually for the medical subdomains is time consuming and costly; therefore, unsupervised distributional methods may be used for identifying these concepts. One output of the mapping and expansion task is the creation of synonyms and abbreviation-expansion pairs. A nice overview of the area can be found in Henriksson et al. (2014).

An approach using distributional semantics for identifying abbreviations and synonyms in Swedish clinical text is described in Henriksson et al. (2014), where a combination of two models, Random Indexing and Random Permutation, was used which outperformed a single model. The results measured for recall were 0.39 for abbreviations to expanded forms (long forms), 0.33 for expanded forms (long forms) to abbreviations and 0.47 for synonyms.

[12]OpenEHR, https://en.wikipedia.org/wiki/OpenEHR. Accessed 2018-01-11.

[13]OpenEHR, http://www.openehr.org. Accessed 2018-01-11.

Skeppstedt et al. (2012) used a rule-based approach to match Swedish SNOMED CT terms to a Swedish clinical text. Spelling correction of the clinical text using Levenshtein distance improved the recall slightly.

An approach for Japanese patient blogs is found in Ahltorp et al. (2014) where the authors obtained promising results for the semantic category *pharmaceutical drug* with a recall of 25% for top *n* candidates.

Alfalahi et al. (2015) used an approach utilising distributional semantics implemented as random indexing to find synonyms in Swedish scientific medical text (Läkartidningen). Clustering was used on the semantic vectors to produce centroids. It was shown that the proximity to the centroid of a number of semantically similar seed words was a successful method for ranking candidate terms from the random indexing algorithm.

For more details on abbreviation detection see Sect. 7.3.4.

## 5.7 Summary of Medical Classifications and Terminologies

This chapter introduced and discussed medical terminologies and standards, the most important being ICD and SNOMED CT specifically used for the patient records but also MeSH for indexing medical literature. ATC classification of drug codes was also introduced; ATC codes describe the chemical components, therapeutic effects and pharmacological class of the drug. All these terminologies and standards are available in several languages.

Different standards for the interoperability of patient record systems were presented. Mapping of terminologies to clinical text was described. The interoperability or mapping of ICD-10 and SNOMED CT will be discussed in Sect. 10.8.8. Other important standards and codes presented were UIMA, FHIR, HL7 and OpenEHR.

**Open Access** This chapter is licensed under the terms of the Creative Commons Attribution 4.0 International License (http://creativecommons.org/licenses/by/4.0/), which permits use, sharing, adaptation, distribution and reproduction in any medium or format, as long as you give appropriate credit to the original author(s) and the source, provide a link to the Creative Commons license and indicate if changes were made.

The images or other third party material in this chapter are included in the chapter's Creative Commons license, unless indicated otherwise in a credit line to the material. If material is not included in the chapter's Creative Commons license and your intended use is not permitted by statutory regulation or exceeds the permitted use, you will need to obtain permission directly from the copyright holder.
